# MiR-4649-5p acts as a tumor-suppressive microRNA in triple negative breast cancer by direct interaction with PIP5K1C, thereby potentiating growth-inhibitory effects of the AKT inhibitor capivasertib

**DOI:** 10.1186/s13058-023-01716-2

**Published:** 2023-10-06

**Authors:** Katharina Jonas, Felix Prinz, Manuela Ferracin, Katarina Krajina, Barbara Pasculli, Alexander Deutsch, Tobias Madl, Beate Rinner, Ondrej Slaby, Christiane Klec, Martin Pichler

**Affiliations:** 1 Division of Oncology, Department of Internal Medicine, Medical University of Graz, Graz, Austria; 2 Research Unit for Non-Coding RNA and Genome Editing in Cancer, Medical University of Graz, Graz, Austria; 3 Department of Experimental, Diagnostic and Specialty Medicine, University of Bologna, Bologna, Italy; 4 Translational Oncology, II. Med Clinics Hematology and Oncology, Augsburg, Germany; 5 Fondazione IRCCS Casa Sollievo della Sofferenza Laboratorio di Oncologia, San Giovanni Rotondo, FG Italy; 6 Division of Hematology, Department of Internal Medicine, Medical University of Graz, Graz, Austria; 7 Division of Molecular Biology and Biochemistry, Gottfried Schatz Research Center for Cell Signaling, Metabolism and Aging, Medical University of Graz, Graz, Austria; 8 BioTechMed-Graz, Graz, Austria; 9 Department for Biomedical Research, Medical University of Graz, Graz, Austria; 10 Department of Biology, Faculty of Medicine and Central European Institute of Technology, Masaryk University, Brno, Czech Republic

**Keywords:** microRNA (miRNA), Triple negative breast cancer (TNBC), Phosphatidylinositol-4-phosphate 5-kinase type 1 gamma (PIP5K1C), AKT signaling, Capivasertib

## Abstract

**Background:**

Triple negative breast cancer (TNBC) is a particularly aggressive and difficult-to-treat subtype of breast cancer that requires the development of novel therapeutic strategies. To pave the way for such developments it is essential to characterize new molecular players in TNBC. MicroRNAs (miRNAs) constitute interesting candidates in this regard as they are frequently deregulated in cancer and contribute to numerous aspects of carcinogenesis.

**Methods and results:**

Here, we discovered that miR-4649-5p, a miRNA yet uncharacterized in breast cancer, is associated with better overall survival of TNBC patients. Ectopic upregulation of the otherwise very low endogenous expression levels of miR-4646-5p significantly decreased the growth, proliferation, and migration of TNBC cells. By performing whole transcriptome analysis and physical interaction assays, we were able to identify the phosphatidylinositol phosphate kinase PIP5K1C as a direct target of miR-4649-5p. Downregulation or pharmacologic inhibition of PIP5K1C phenocopied the growth-reducing effects of miR-4649-5p. PIP5K1C is known to play an important role in migration and cell adhesion, and we could furthermore confirm its impact on downstream PI3K/AKT signaling. Combinations of miR-4649-5p upregulation and PIP5K1C or AKT inhibition, using the pharmacologic inhibitors UNC3230 and capivasertib, respectively, showed additive growth-reducing effects in TNBC cells.

**Conclusion:**

In summary, miR-4649-5p exerts broad tumor-suppressive effects in TNBC and shows potential for combined therapeutic approaches targeting the PIP5K1C/PI3K/AKT signaling axis.

**Supplementary Information:**

The online version contains supplementary material available at 10.1186/s13058-023-01716-2.

## Background

Breast cancer is the type of cancer with the highest incidence rate in women worldwide [1]. It is important to bear in mind though that breast cancer is not just one type of malignancy but comprises several very heterogenous subtypes in terms of biology as well as clinical behavior and treatment algorithms [2]. And while triple negative breast cancer (TNBC) only accounts for around 15–20% of all breast cancer cases, it is the subtype with the highest mortality rate [2, 3]. This is owed to several facts. First, TNBC is particularly aggressive and often characterized by a high proliferation rate, high metastatic capability, rapidly developing resistance to chemotherapy, and thus a high rate of recurrence [2–4]. In addition, TNBC lacks the expression of the estrogen receptor (ER), progesterone receptor (PR), and human epidermal growth receptor 2 (HER2), making it difficult to treat due to the absence of these therapeutic targets [1–3]. It is thus of high interest to identify novel factors contributing to TNBC in order to develop new therapeutic strategies.

In doing so, a promising approach may be found in investigating non-coding RNAs (ncRNAs), as they were described to function as important players in malignancies, including breast cancer [5–8]. The term ncRNA encompasses a large variety of non-protein-coding RNA molecules that fulfill a multitude of regulatory roles [5, 8–10]. Among them, microRNAs (miRNAs), a conserved class of short ncRNAs with an average length of around 22 nucleotides, often show altered expression patterns in cancer which contribute to diverse aspects of cancer initiation, progression, and recurrence [6, 7, 11, 12]. In breast cancer, for example, numerous miRNAs were discovered to affect proliferation, migration, invasion, metastasis, and stemness [13–16]. MiRNAs exert these effects by targeting large numbers of mRNAs via complementary base pairing to a short seed region, mostly within the 3' untranslated region (UTR) of the mRNA, which entails degradation or translational inhibition of the mRNA by means of the RNA-induced silencing complex (RISC) [11, 17–19].

Considering the fact that TNBC is still a particularly challenging type of malignancy due to the lack of therapeutic targets and the fact that miRNAs have been proven to be critical players in cancer, which also paved the way for miRNA mimetics and inhibitors in clinical trials [20, 21], we aimed to identify a novel miRNA with potential therapeutic usefulness in TNBC. To this end, we selected a still uncharacterized miRNA in breast cancer, called miR-4649-5p, which was detected in a previous study employing an ncRNA microarray screen of spheroids generated from breast cancer cell lines [13]. This miRNA has recently been associated with prognosis in patients with B-cell acute lymphoblastic leukemia (B-ALL) [22].

To find out whether miR-4649-5p plays a role in the pathogenesis of TNBC, we determined its endogenous expression levels in TNBC cell lines and correlated expression with patient prognosis. Based on the observation that the expression levels were very low, we tested the hypothesis that ectopic upregulation of miR-4649-5p could have tumor-suppressive effects and observed reduced growth, proliferation, and migration of the cells. In addition, we identified a direct target of miR-4649-5p and a signaling pathway downstream of this target, which helps to explain the tumor-suppressive properties of miR-4649-5p. Lastly, this enabled us to test combinatorial treatments of miR-4649-5p upregulation and downstream pathway inhibition, which showed additive growth-reducing effects in vitro.

## Methods

### Patient cohorts and in silico analysis

To assess the prognostic significance of miR-4649-5p and PIP5K1C in breast cancer patients, an online publicly available Kaplan–Meier plotter was used that provides separate specialized software tools for analyzing the impact of microRNAs, mRNA, or protein on the survival of breast cancer patient cohorts originating from different databases (https://kmplot.com/analysis/index.php?p=service&cancer=breast_mirna [23] for miR-4649-5p; https://kmplot.com/analysis/index.php?p=service&cancer=breast [24] for PIP5K1C on the RNA level, and https://kmplot.com/analysis/index.php?p=service&cancer=breast_protein [25] for PIP5K1C on the protein level). For miR-4649-5p, data on the overall survival of 97 patients with triple negative breast cancer (TNBC) was available from The Cancer Genome Atlas Project (TCGA) [23]. For PIP5K1C the overall survival of 153 TNBC patients as well as the relapse-free survival of 392 TNBC patients was analyzed based on gene chip data originating from the Gene Expression Omnibus (GEO) database [24], and the overall survival of 65 breast cancer patients without subtype discrimination was analyzed based on proteomics data [25]. No subtype discrimination could be applied in this case, as the cohort size was too small to limit it to TNBC patients. For all survival analyses, a follow-up threshold of 60 months was set and patients were split into high and low expression groups by an auto-selected optimal cutoff. The online analysis tool TNMplot [26] was employed to analyze the expression of PIP5K1C in invasive breast carcinomas (n = 112) compared to paired healthy tissue (n = 112) from RNA-seq-based TCGA data.

### Cell lines and cell culture conditions

The following triple negative breast cancer cell lines were used in this study: SUM159, MDA-MB-231, and BT-20. MDA-MB-231 and BT-20 cells were obtained from the American Type Culture Collection (ATCC; Manassas, CA, USA). SUM159 cells were purchased from Asterand (Detroit, MI, USA). Furthermore, HEK239 cells purchased from the American Type Culture Collection (ATCC) were used.

MDA-MB-231 and HEK239 cells were maintained in high-glucose DMEM (4.5 g/l d-Glucose, l-Glutamine, 25 mM HEPES; Gibco, Thermo Fisher Scientific, Waltham, MA, USA), 10% fetal bovine serum (FBS) (Serana, Pessin, Germany), and 1% penicillin/streptomycin (final concentration penicillin: 100 units/ml, final concentration streptomycin: 100 µg/ml; Sigma-Aldrich, St. Louis, MO, USA). SUM159 cells were grown in Ham's Nutrient Mixture F12 containing 1 mM l-Glutamine (GE Healthcare Life Sciences, Pittsburgh, USA) supplemented with 2 mM HEPES buffer (Gibco), 5 μg/ml insulin (Sigma-Aldrich), 1 μg/ml hydrocortisone (Sigma-Aldrich), 1% penicillin/streptomycin (Sigma-Aldrich), and 5% FBS (Serana). BT-20 were cultivated in Minimum Essential Medium—Eagle with Earle's BSS and l-Glutamine (Lonza, Basel, Switzerland) supplemented with 10% FBS (Serana) and 1% penicillin/streptomycin (Sigma-Aldrich). All cell lines were cultivated in a 5% CO_2_ humidified incubator at 37 °C. SUM159 and MDA-MB-231 cells were tested for mycoplasma with the Venor GeM Mycoplasma Detection Kit (Minerva Biolabs, Berlin, Germany) by the Core Facility for Alternative Biomodels and Preclinical Imaging of the Medical University of Graz, Austria.

### Transient miR-4649-5p mimic and PIP5K1C siRNA transfection

To achieve transient overexpression of miR-4649-5p or knockdown of PIP5K1C, TNBC cell lines were transfected with 10 nM mirVana™ hsa-miR-4649-5p mimic or mirVana™ mimic control (Thermo Fisher Scientific) and 20 nM PIP5K1C siRNA #8 (targeting sequence: TTCCTGTACTGTAAAGACTAA; Qiagen) or AllStars Negative Control siRNA (Qiagen), respectively.

Cells in 6-well plates or 6 cm dishes were transfected using the HiPerFect Transfection Reagent (Qiagen) following the fast-forward transfection protocol of the manufacturer. Cells in 96-well plates were transfected according to the reverse transfection protocol. To confirm efficient overexpression or knockdown 48 h after transient transfection, quantitative RT-PCR was applied.

### Generation of stable miR-4649-5p mimic cells by lentiviral transduction

SUM159 cells were seeded and incubated overnight in complete growth medium in a 24-well plate. After 24 h, medium was replaced with complete growth medium containing ViralPlus Transduction Enhancer (ABM, Richmond, BC, Canada) diluted 1:200 and 10 μg/ml polybrene (Santa Cruz Biotechnology, Santa Cruz, CA, USA). Cells were transduced by dropwise addition of 10 μl of miR-4649-5p mimic virus (LentimiRa-GFP-hsa-miR-4649-5p Virus, ABM) or control virus (Lenti-III-mir-Off Control Virus, ABM). 48 h after transduction, cells were selected with 1.5 μg/ml puromycin dihydrochloride (Gibco) for 1 week while monitoring GFP expression by fluorescent microscopy. Subsequently, cells were sorted by FACS for high GFP expression. Overexpression of miR-4649-5p was checked by quantitative RT-PCR.

### RNA isolation and cDNA synthesis for quantitative RT-PCR

RNA was isolated from cell lines at a confluency of 75–95% in biological triplicates using TRIzol™ Reagent (Thermo Fisher Scientific) following the manufacturer`s protocol. For quantitative RT-PCR, 1 μg of total RNA per sample was reverse transcribed into cDNA either with the miScript II RT Kit (Qiagen) using the miScript HiFlex Buffer for the simultaneous detection of miRNA and mRNA, or the QuantiTect Reverse Transcription Kit (Qiagen) for the detection of only mRNA, both according to the manufacturer’s protocols.

### Quantitative RT-PCR (RT-qPCR)

Quantitative RT-PCR was carried out in technical duplicates using the QuantiTect SYBR Green PCR Kit (Qiagen) according to the manufacturer’s two-step RT-PCR protocol. To measure miR-4649-5p expression, the Hs_miR-4649-5p_1 miScript Primer Assay (Qiagen) was used together with the miScript Universal Primer from the miScript SYBR Green PCR Kit (Qiagen) and was normalized to the two housekeepers SNORD61 and SNORD95 using the Hs_SNORD61_11 and Hs_SNORD95_11 miScript Primer Assays (Qiagen) according to the manufacturer`s instructions.

For the detection of coding genes together with the housekeepers GAPDH and U6, primers were designed with the NIH Primer Blast Tool (https://www.ncbi.nlm.nih.gov/tools/primer-blast/) and purchased from Eurofins Scientific. A list of these primer sequences is given in Additional file 1: Table S1.

Per qPCR reaction (volume of 10 µl), 1 ng of cDNA was used. The measurements were carried out in LightCycler® 480 Multiwell Plates 384 on a LightCycler® 480 Real-Time PCR System (Roche, Basel, Switzerland). Ct values were normalized by subtracting the respective arithmetic mean of the two housekeeper genes to receive delta Ct (ΔCt) values. Following the ΔΔCt method, relative expression levels were calculated by subtracting the ΔCt of the respective negative control and were plotted as 2^−ΔΔCt^.

### Digital droplet PCR (ddPCR)

To determine miR-4649-5p expression by ddPCR, RNA was isolated using TRIzol™ (Thermo Fisher Scientific) as indicated by the manufacturer and 10 ng total RNA were reverse transcribed with the TaqMan Advanced miRNA cDNA Synthesis Kit (Thermo Fisher Scientific) according to the protocol. cDNA was diluted 1:100 and 5 μl were used per 20 μl reaction with 10 μl ddPCR Supermix for Probes (No dUTP; BioRad) and 0.5 μl TaqMan Advanced miRNA Assay for miR-4649-5p (Thermo Fisher Scientific). Droplets were generated from the samples in duplicates with a QX200™ droplet generator (BioRad) using Droplet Generation Oil for Probes (BioRad) and DG8™ Cartridges for QX200™ (BioRad) according to the manufacturer. Droplets were transferred into semi-skirted 96-well ddPCR plates (BioRad), plates were heat-sealed, and PCR was performed on a T100TM Thermal Cycler (BioRad) (95 °C 10 min; 95 °C 30 s + 61 °C 1 min × 39; 98 °C 10 min; ramping 2 °C/s). The droplets were read on a QX200™ droplet reader device (BioRad) and data were evaluated using the QX Manager Software Version 1.2 (BioRad).

### WST-1 cell growth assay

To assess the impact of miR-4649-5p and PIP5K1C on cellular growth, 3 × 10^3^ SUM159, 5 × 10^3^ MDA-MB-231, and 6 × 10^3^ BT-20 cells were seeded per well of a 96-well plate (one plate for each time point) and transiently transfected with the miRNA mimic, PIP5K1C siRNA and their respective controls in six replicates using HiPerFect Transfection Reagent (Qiagen) according to the reverse transfection protocol of the manufacturer. To investigate potential additive effects of miR-4649-5p mimic and PIP5K1C or AKT inhibition, cells were additionally treated with 10 μM of the PIP5K1C inhibitor UNC3230 (MedChemExpress, Monmouth Junction, NJ, USA), 0.5 μM of the AKT inhibitor capivasertib (MedChemExpress) or equal volumes of DMSO as vehicle control. Cells were incubated for 24, 48, 72, and 96 h. At each time point, WST-1 proliferation reagent (Roche) was added to the wells in a 1:10 ratio according to the manufacturer’s instructions. After incubation at 37 °C for 60 or 120 min (depending on the cell line), colorimetric changes were measured using a SPECTROstar Omega (BMG Labtech, Ortenberg, Germany) at a wavelength of 450 nm with a reference wavelength of 620 nm.

### Colony formation assay

To confirm the impact of altered miR-4649-5p and PIP5K1C expression on cell growth over a longer time frame, we performed clonogenic assays. Transiently transfected cells were trypsinized 24 h after transfection, counted, and seeded in 6-well plates with 200 (SUM159) or 500 (MDA-MB-231) cells/well. Cells were cultured at 37 °C and 5% CO_2_. After 7 (SUM159) or 14 days (MDA-MB-231), cells were fixed and stained with 0.04% crystal violet (Sigma-Aldrich) in 20% methanol/PBS. The number of colonies was counted macroscopically. Each experiment was carried out in triplicates or sextuplicates.

### Protein extraction and western blotting

Total proteins were isolated from fresh cells using Radioimmunoprecipitation Assay (RIPA) buffer (Sigma-Aldrich) supplemented with 1:50 protease inhibitor cocktail P8340 (Sigma-Aldrich). From each sample, 25 µg of protein were diluted in 4× Laemmli buffer (BioRad, Hercules, CA, USA) containing 10% β-mercaptoethanol (Sigma-Aldrich) and separated by sodium dodecyl sulfate polyacrylamide gel electrophoresis (SDS-PAGE) on a 4–15% Mini-PROTEAN® TGX™ Precast Gel (BioRad) before plotting on nitrocellulose membranes (BioRad). Membranes were blocked for at least 1 h with 5% non-fat milk powder in 1× Tris-buffered saline (TBS; BioRad)/0.1% Tween-20 (Sigma-Aldrich) before incubation with the primary antibodies overnight at 4 °C. Primary antibodies were diluted in 5% BSA (Sigma-Aldrich)/TBS-Tween (BioRad). The following primary antibodies were used: PIP5K1C (#3296, Cell Signaling Technology, Cambridge, United Kingdom) diluted 1:1000, AKT (#9272, Cell Signaling Technology) diluted 1:1000, Phospho-Akt (S473, D9E, #4060, Cell Signaling Technology) diluted 1:2000, and the housekeeper Cofilin (ab42824, Abcam, Cambridge, United Kingdom) diluted 1:10,000. After primary antibody incubation, membranes were washed (three times for 10 min each in TBS-Tween), incubated with the secondary antibody (HRP-conjugated anti-rabbit; diluted 1:1000 in 5% milk/TBS-Tween; Santa Cruz Biotechnology, Dallas, TX, USA) for 1 h and washed again. Signals were detected with an enhanced chemiluminescence detection system (SuperSignal™ West Pico PLUS Chemiluminescent Substrate, Thermo Fisher Scientific) on a BioRad ChemiDoc Touch device. Densitometric quantifications were performed using the Image Lab Software (BioRad). For reprobing, membranes were stripped with 10% acetic acid for 1 h.

### EdU proliferation assay

Cell proliferation was assessed in a flow cytometric assay using the Click-iT™ Plus EdU Pacific Blue™ Flow Cytometry Assay Kit (Thermo Fisher Scientific). Cells were seeded and transfected in 6 cm dishes using HiPerfect (Qiagen) as stated previously. Cells were labeled with 10 µM EdU in full growth medium for 2 h at 37 °C and 5% CO_2_. All subsequent steps were performed according to the Click-iT™ Plus EdU Pacific Blue™ Flow Cytometry Assay protocol. The samples were measured on a CytoFLEX SI (Beckman Coulter) recording 20,000 events per sample. Cell populations were gated to exclude debris/dead cells (R1) and cell aggregates (R2).

### Caspase 3/7 activity assay

To analyze the induction of apoptosis, the activity of effector caspase-3 and -7 was measured using a Caspase-Glo® 3/7 assay (Promega, Madison, WI, USA) according to the manufacturer’s instructions. Cells were transiently transfected in four technical replicates in 96-well plates with 3 × 10^3^ SUM159 and 5 × 10^3^ MDA-MB-231 cells per well using HiPerFect Transfection Reagent (Qiagen) according to the reverse transfection protocol. After 48 h, the luminogenic reagent was added to the cells as instructed in the protocol, and signals were measured with a LUMIstar Omega (BMG LabTech).

### Cell migration assays

Two types of cell migration assays were conducted. First, wound healing assays, also called scratch assays, for which SUM159 cells were seeded and transiently transfected in 6-well plates in four biological replicates. To reach confluence, high cell numbers were seeded (5 × 10^5^) and scratches were introduced after 24 h. Medium was changed and cells were washed to remove scratched-off cells. Closure of the scratches was observed under the microscope after 15/20 h, 24 h, and 45 h. The area of the scratches was determined using the ImageJ (NIH, Bethesda, Maryland) plugin “MRI Wound Healing Tool” and for each time point, the remaining area relative to the 0 h time point was calculated.

To confirm the results of the wound healing assays, transwell migration assays were performed using transwell inserts with 0.4 µm pore size (Corning Incorporated, Corning, NY, USA). Cells were transiently transfected in 6-well plates and after 24 h medium was changed to start starvation without FBS. After 24 h of starvation, cells were seeded on transwell membranes (1.5 × 10^4^ cells for SUM159, 2.5 × 10^4^ cells for MDA-MB-231). The membranes were previously coated with 0.1% gelatin (Sigma-Aldrich) in 0.02 M acetic acid, left to dry overnight, and rehydrated with FBS-free medium at 37 °C for 1 h before seeding the cells in FBS-free medium. The lower chambers were filled with full growth medium containing FBS. After 48 h, cells were fixed with cold methanol and stained with 0.2% crystal violet (Sigma-Aldrich) in 2% ethanol. After washing, cells that did not migrate through the membrane were removed. From each transwell, microscopic images of five representative areas were taken at 40× magnification and counted manually.

### Transcriptome analysis and identification of potential miR-4649-5p targets

For whole transcriptome analysis by RNA-seq, total RNA was isolated from fresh cell pellets with the RNeasy Mini Kit with DNAse treatment according to the manufacturer’s instructions (Qiagen). After quality control of the RNA with an Agilent RNA 6000 Nano Kit on an Agilent 2100 BioAnalyzer system (Agilent Technologies, Santa Clara, CA, USA) and quantification on a NanoDrop 2000 Spectrophotometer (Thermo Fisher Scientific), 250 ng of total RNA were used for library preparation with the NEBNext® rRNA Depletion Kit v2 in combination with the NEBNext® Ultra™ II Directional RNA Library Prep Kit for Illumina® (New England Biolabs GmbH, Frankfurt am Main, Germany) according to manufacturer’s instructions. Libraries were QC checked with an Agilent 2100 DNA high sensitivity kit, pooled, and sent to Vienna BioCenter Core Facilities GmbH (Vienna, Austria) for sequencing in an Illumina NovaSeq SP flow cell (Illumina, Eindhoven, Netherlands) in SR100 mode. After demultiplexing, FASTQ files were used for data analysis.

To identify potential miRNA targets, significantly downregulated genes were determined based on *p* values adjusted for multiple testing and ranked according to their log2-fold change values. In silico target predictions were performed for the top candidates using the online tools TargetScan [27], miRWalk2.0 [28], and miRDB [29].

A Gene Ontology (GO) enrichment analysis was performed on significantly downregulated genes using the PANTHER Overrepresentation Test (http://www.pantherdb.org/; GO Ontology database https://doi.org/10.5281/zenodo.6799722, released 2022-07-01, reference list Homo sapiens, annotation data set GO biological process complete, Fisher’s Exact Test).

### Dual luciferase reporter assay

To confirm the direct interaction of miR-4649-5p with the putative target PIP5K1C, a 60 nt region of the 3′ UTR of PIP5K1C containing a predicted binding site for the miRNA was inserted into the dual luciferase reporter vector pEZX-MT06 (Genecopoeia, Rockville, MD, USA). Both the wild-type (5′ CCCCAAACACTGGTTTGCATCCCAGGTTCCTCGCCCACCTACCCCCGCCACACCCCGTCT 3′) and a mutated binding site sequence (5′ CCCCAAACACTGGTTTGCATCGTAGGTTCCAGGTTCACCTACCCCCGCCACACCCCGTCT 3′) were used. An empty control plasmid (CmiT000001-MT06; Genecopoeia) was employed as a reference control. For the luciferase assay, HEK293 cells were seeded in 24-well plates. Once cells reached 70–80% confluence they were co-transfected with 200 ng pEZ-MT06 PIP5K1C wt/mutated reporter vector or control vector and 50 nM mirVana™ miR-4649-5p mimic or mirVana™ mimic control (Thermo Fisher Scientific) using Lipofectamine 2000 Transfection Reagent (Thermo Fisher Scientific) and Opti-MEM Reduced Serum Medium (Thermo Fisher Scientific) according to the manufacturer’s instructions. Cells were harvested 24 h after transfection and the Luc-Pair Luciferase Assay Kit 2.0 (Genecopoeia) was performed according to the user manual. Luminescence was measured with a LUMIStar Omega luminometer (BMG LabTech). The firefly luciferase signals were normalized by the renilla luciferase signals.

### Statistical analysis

Statistical analysis was performed using GraphPad Prism Version 5.01 (GraphPad Software, Inc., San Diego, CA, USA). Differences between transfected samples and respective controls were assessed by unpaired, two-tailed independent t-tests with a 95% confidence interval, unless indicated otherwise. *p* values below 0.05 were considered statistically significant (*p < 0.05, **p < 0.01, ***p < 0.005).

## Results

### MiR-4649-5p is associated with better survival of TNBC patients and shows very low endogenous expression in TNBC cell lines

**Fig. 1:**
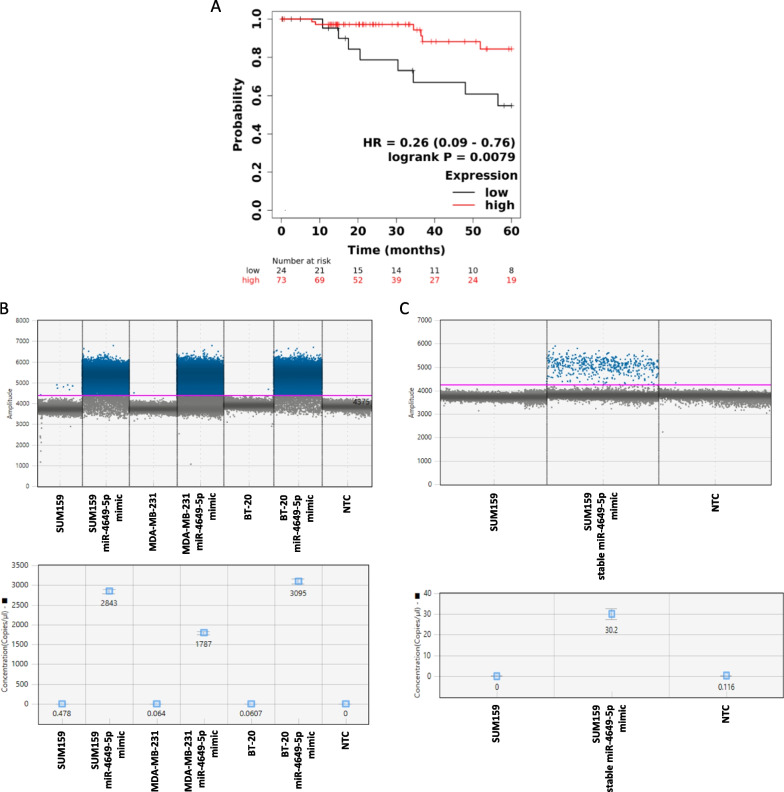
MiR-4649-5p is associated with better patient survival and shows low expression in triple negative breast cancer cell lines. **A** Kaplan Meier analysis showing overall survival of patients with TNBC split into a miR-4649-5p low (in black; n = 24) and high (in red; n = 73) expression group (log-rank test; *p* = 0.0079; Hazard Ratio (HR) = 0.26; confidence interval 0.09–0.76). **B** Digital droplet PCR showing endogenous miR-4649-5p expression in SUM159, MDA-MB-231, and BT-20 cells and transient miR-4649-5p mimic overexpression as positive controls. Droplet distributions including the threshold for positive droplets (top) and quantifications giving miR-4649-5p copies per μl (bottom) are shown (NTC…no-template control). **C** Digital droplet PCR showing endogenous expression and stable miR-4649-5p overexpression in SUM159 cells. Droplet distributions including the threshold for positive droplets (top) and quantifications giving the copies of miR-4649-5p per μl (bottom) are depicted (NTC…no-template control)

In order to determine whether miR-4649-5p plays a role in the carcinogenesis or progression of triple negative breast cancer (TNBC), we explored its clinical relevance in a patient cohort. Kaplan Meier analysis of a dataset originating from The Cancer Genome Atlas Project (TCGA) revealed that higher expression levels of the *miR-4649* gene are associated with significantly better overall survival (OS) of patients with TNBC (log-rank test; *p* = 0.0079; Hazard Ratio (HR) = 0.26; 95% confidence interval 0.09–0.76) (Fig. 1A). In this context, it has to be noted that the overall median expression level in the RNA-seq-based TCGA data set was very low.

Next, we aimed to determine the expression levels of miR-4649-5p in three different TNBC cell lines using an absolute quantification approach by digital droplet PCR (ddPCR). As positive controls, to confirm the specificity of the ddPCR probes, we generated an ectopic overexpression of miR-4649-5p by transient mimic transfection in three TNBC cell lines, as well as a stable lentivirus-based overexpression in one of the cell lines (SUM159). We observed that there was close to zero endogenous expression detectable in all three cell lines, whereas the positive controls showed a strong (in case of transient mimic transfection) to medium (in case of stable mimic transfection) increase in positive droplets (Fig. 1B, C).

In order to independently confirm the low expression of miR-4649-5p in the TNBC cell lines, we employed a quantitative RT-PCR (RT-qPCR) approach, again using cells with a transient or stable overexpression as positive controls. Looking at the melting curves of these PCR assays we found that the strong transient miR-4649-5p mimic overexpression (approximately 15-fold; Additional file 2: Fig. S1A) allowed specific amplification of the miRNA, confirming specificity of the used primers, whereas the lack of endogenous miR-4649-5p in the cell lines led to low specific peaks and mostly resulted in the amplification of unspecific products (as visible by multiple peaks in the melting curves) (Additional file 2: Fig. S1B). The medium overexpression of miR-4649-5p that was achieved by stable transfection (approximately twofold; Additional file 2: Fig. S1A), did still result in some unspecific amplification, but less so than in the case of the barely present endogenous miR-4649-5p (Additional file 2: Fig. S1B). This shows that the lower the expression of miR-4649-5p, the more unspecific amplification occurs.

### MiR-4649-5p exerts tumor-suppressive effects in TNBC cells by reducing their growth, proliferation, and migration

**Fig. 2:**
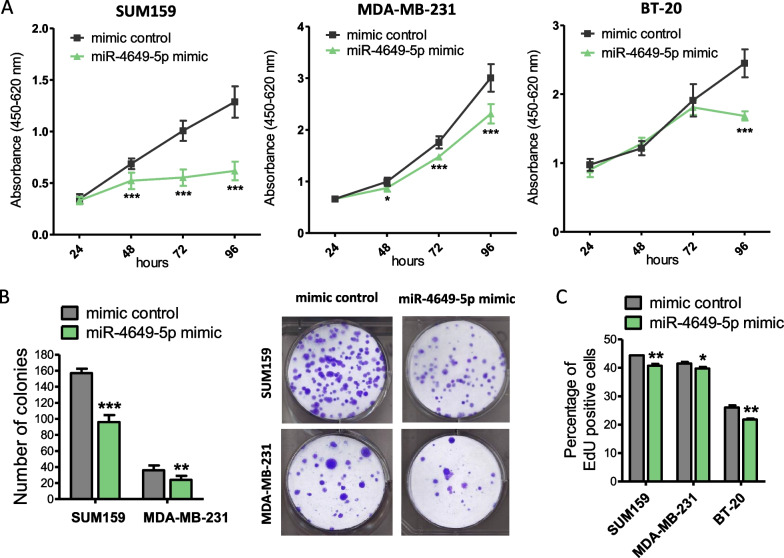
MiR-4649-5p reduces growth and proliferation of TNBC cell lines. **A** The effect of transient miR-4649-5p mimic transfection on cell growth was assessed by WST-1 assays in three TNBC cell lines (n = 6; mean ± SD; *p ≤ 0.05, ***p ≤ 0.005). **B** SUM159 and MDA-MB-231 cells were transiently transfected with miR-4649-5p mimic or mimic control and seeded at low density to observe colony formation after 7 (SUM159) or 14 days (MDA-MB-231). Absolute numbers of colonies were counted (as seen in the representative images on the right) and are presented as mean ± SD (on the left) (n = 3; **p ≤ 0.01, ***p ≤ 0.005). **C** Cell proliferation was assessed 72 h after transient miR-4649-5p mimic transfection in a flow cytometric EdU assay (n = 3; mean ± SD; *p ≤ 0.05, **p ≤ 0.01)

As we had discovered that miR-4649-5p exhibits very low expression levels in three TNBC cell lines and that higher expression in tumor tissue is associated with improved survival of TNBC patients, we became interested in exploring whether ectopic upregulation of the miRNA could have tumor-suppressive effects in TNBC, thus presenting a possible therapeutic opportunity. In in vitro growth assays over 96 h we observed that transient miR-4649-5p mimic overexpression resulted in decreased growth of all three TNBC cell lines (Fig. 2A). To confirm the impact of miR-4649-5p on cellular growth over a longer period, we performed colony formation assays which showed a decrease in SUM159 and MDA-MB-231 colonies upon miR-4649-5p overexpression (Fig. 2B) (the BT-20 cell line did not form colonies under these conditions). To further elucidate the mechanism behind this growth phenotype, we investigated cell proliferation in a flow cytometric EdU assay and observed lower percentages of proliferating cells upon miR-4649-5p mimic transfection (Fig. 2C; Additional file 2: Fig. S2), both corroborating and explaining the decreased cellular growth (Fig. 2A).

In addition, we tested the possibility of cell death induction by miR-4649-5p upregulation but could not detect any changes in caspase 3/7 activity (Additional file 2: Fig. S3), indicating that the differences observed in the growth assays were primarily due to the decrease in proliferation and not due to reduced cell numbers caused by apoptosis.

**Fig. 3:**
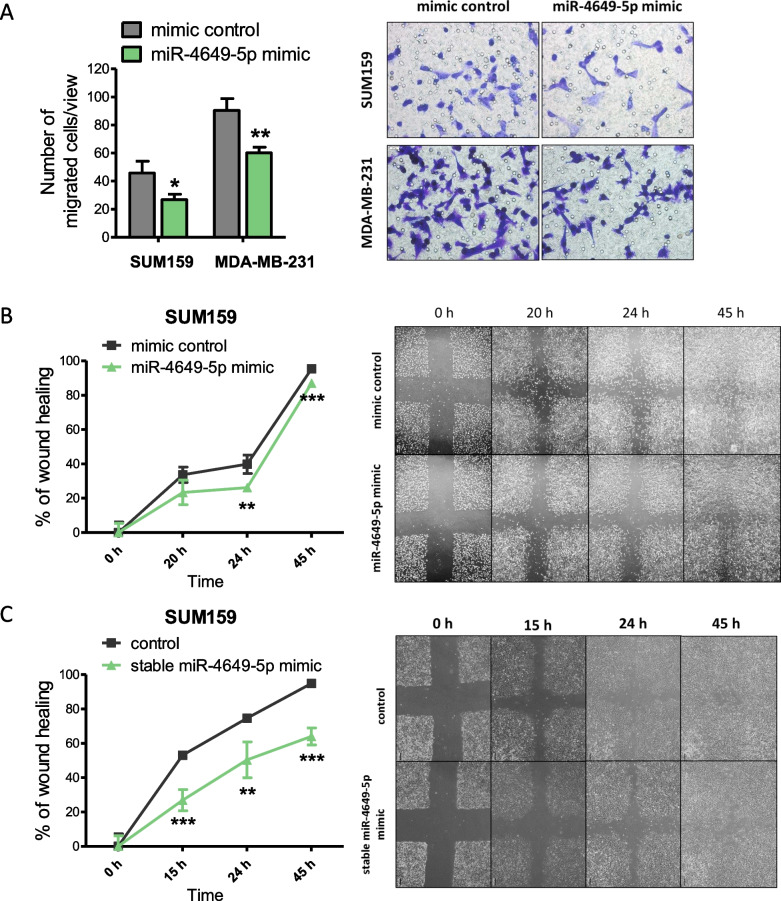
MiR-4649-5p reduces migration of TNBC cell lines. **A** The effect of transient miR-4649-5p mimic transfection on cell migration was assessed in a transwell migration assay. From each transwell, the number of cells in five representative fields of view (as seen in the representative pictures on the right) were counted and are presented as mean ± SD (n = 3; *p ≤ 0.05, **p ≤ 0.01). The impact of **B** transient and **C** stable miR-4649-5p overexpression on the migration of SUM159 cells was confirmed in scratch assays, where the closure of a scratch was observed for up to 45 h (as seen in the representative images on the right). At each time point the scratch areas relative to the 0 h time point were calculated and are presented as curves showing the progress of wound closure over time (n = 4; mean ± SD; **p ≤ 0.01, ***p ≤ 0.005)

We then continued by investigating the impact of miR-4649-5p on cell migration, another important characteristic of cancer cells that enables them to facilitate tumor progression. To study migration, we performed both transwell migration assays as well as scratch assays. In the transwell migration assays, transient miR-4649-5p mimic transfection of SUM159 and MDA-MB-231 TNBC cells caused a significant reduction in the number of cells that migrated through the permeable membrane (Fig. 3A). Scratch assays with transient and stable miR-4649-5p mimic overexpressing SUM159 cells showed a significant delay in the closure of a scratch introduced in a confluent cell layer (Fig. 3B, C), also confirming reduced migration.

In summary, ectopic upregulation of the otherwise very low/non-detectable expression of miR-4649-5p in TNBC cell lines negatively impacts the growth, proliferation, and migration of the cancer cells, outlining a tumor-suppressive role and giving the possibility for a therapeutic application of the miRNA in TNBC.

### Phosphatidylinositol-4-phosphate 5-kinase type 1 gamma (PIP5K1C) is a direct target of miR-4649-5p and influences the growth of TNBC cells

**Fig. 4:**
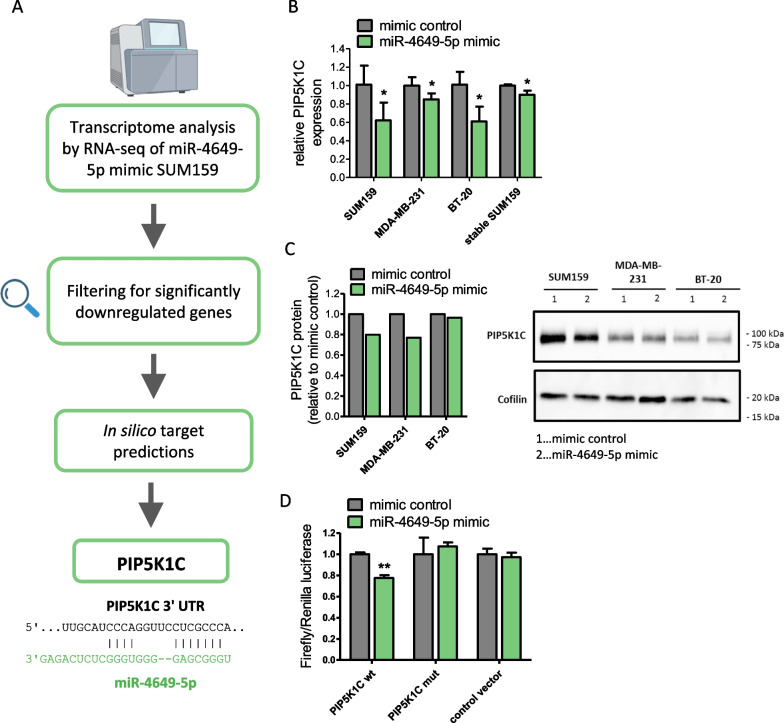
Phosphatidylinositol-4-phosphate 5-kinase type 1 gamma (PIP5K1C) is a direct target of miR-4649-5p. **A** Scheme illustrating the identification of PIP5K1C as a putative target of miR-4649-5p by RNA-seq analysis of SUM159 miR-4649-5p mimic transfected cells and subsequent in silico target predictions. At the bottom, a target sequence between miR-4649-5p and PIP5K1C is presented. **B** The downregulation of PIP5K1C by the miR-4649-5p mimic was confirmed in three transiently transfected (48 h) and one stable TNBC cell line by RT-qPCR (n = 3; mean ± SD; *p ≤ 0.05). **C** The downregulation of PIP5K1C protein by the miR-4649-5p mimic was confirmed 48 h after transduction in three TNBC cell lines by Western Blotting. Densitometric quantification of the representative blot on the right, normalized to the housekeeper Cofilin, is presented on the left. **D** A dual luciferase reporter assay giving evidence for the direct binding of the miR-4649-5p mimic to a reporter construct carrying a wild type (wt) target sequence of the PIP5K1C 3' UTR which was compared to an empty control vector. Binding of the miRNA, and thus luciferase signal reduction, was abolished when the PIP5K1C sequence was mutated (mut) (n = 3; mean ± SD; **p ≤ 0.01)

Since miR-4649-5p had shown tumor-suppressive effects in TNBC cells, we sought to unravel underlying molecular mechanisms by identifying targets of the miRNA. Knowing which targets or pathways are responsible for the tumor-suppressive properties might also allow to test already existing treatment options against these targets in combination with miR-4649-5p upregulation or to identify novel vulnerabilities of TNBC. To this end, we performed an RNA-seq-based whole transcriptome analysis of SUM159 cells transiently transfected with the miR-4649-5p mimic. The analysis revealed 433 genes to be significantly differentially expressed (238 upregulated and 195 downregulated) compared to control transfected cells (for the unfiltered RNA-seq results see Additional file 3). In the next step, we filtered the results to only focus on genes that were downregulated and could thus be potential direct targets of miR-4649-5p. Of the 195 genes that were downregulated, 52 annotated genes showed a more than 1.5-fold downregulation. For the top 20 of these downregulated genes (presented in Additional file 1: Table S2), in silico target predictions were performed with three different online tools (TargetScan [27], miRWalk2.0 [28], and miRDB [29]) to search for potential miR-4649-5p binding sites. For those putative targets, where binding sites were predicted by at least two of the three tools, literature research was performed to select candidates that might explain the phenotype caused by miR-4649-5p. A scheme illustrating the steps taken to pick out potential miRNA targets is presented in Fig. 4A.

Following these steps, a promising target turned out to be the phosphatidylinositol-4-phosphate 5-kinase type 1 gamma (PIP5K1C) (sequences of the two predicted miR-4649-5p binding sites in the PIP5K1C 3' UTR are presented in Additional file 1: Table S3). PIP5K1C, also referred to as PIP5Kγ, is a kinase that facilitates the production of phospholipid second messengers that are critically involved in fundamental cellular processes like cell migration and cell growth [30, 31]. Thus, downregulation of PIP5K1C by miR-4649-5p, and consequent downregulation of its downstream products, could explain the tumor-suppressive effects of miR-4649-5p. We confirmed the downregulation of PIP5K1C by miR-4649-5p on mRNA (Fig. 4B) and protein level (Fig. 4C) in different TNBC cell lines by RT-qPCR and Western Blotting, respectively. To prove that miR-4649-5p downregulates PIP5K1C by directly interacting with it, we performed a dual luciferase reporter assay. We observed that co-transfection of the miR-4649-5p mimic and the luciferase reporter construct carrying the predicted binding site 2 of the PIP5K1C 3' UTR (Additional file 1: Table S3) led to a reduction in the luciferase signal, which was not the case when the binding sequence was mutated by exchange of 6 nucleotides (Fig. 4D).

**Fig. 5:**
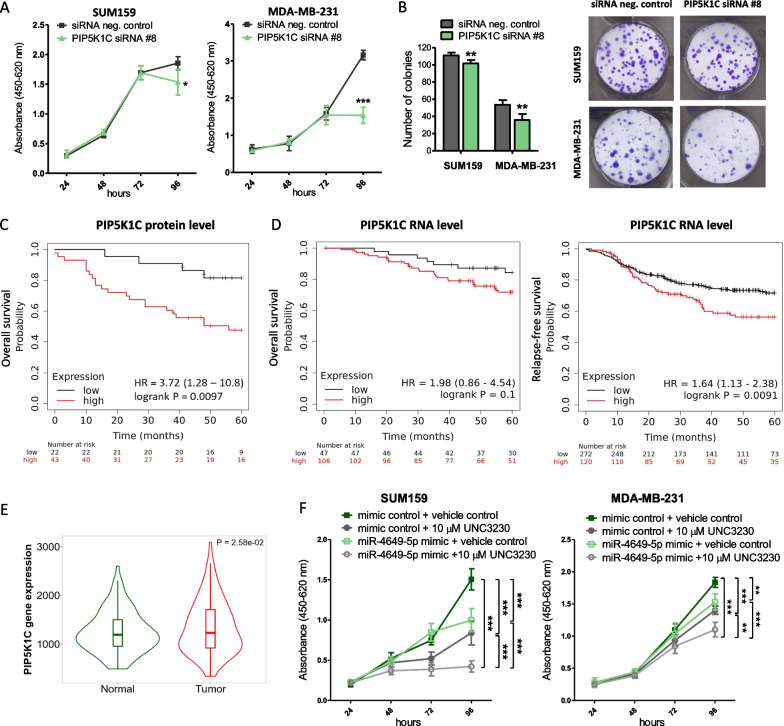
Phosphatidylinositol-4-phosphate 5-kinase type 1 gamma (PIP5K1C) knockdown or pharmacologic inhibition reduces TNBC growth. **A** The effect of siRNA-mediated PIP5K1C knockdown on cell growth of two TNBC cell lines was assessed in WST-1 assays (n = 6; mean ± SD; *p ≤ 0.05; ***p ≤ 0.005). **B** SUM159 and MDA-MB-231 cells were transiently transfected with 20 nM PIP5K1C siRNA or control and seeded at low density to observe colony formation after 7 (SUM159) or 14 days (MDA-MB-231). Absolute numbers of colonies (as seen in the representative images on the right) were counted and are presented as mean ± SD (on the left) (n = 6; **p ≤ 0.01). **C** Overall survival of breast cancer patients split into a PIP5K1C low (black; n = 19) and high (red; n = 46) group based on protein levels from proteomic data (log-rank test; *p* = 0.0097; Hazard Ratio (HR) = 3.72; confidence interval 1.28–10.8). **D** Overall survival of TNBC patients split into a PIP5K1C low (black; n = 47) and high (red; n = 106) expression group (log-rank test; *p* = 0.1; Hazard ratio (HR) = 1.98; confidence interval 0.86 – 4.54) and relapse-free survival of TNBC patients split into a PIP5K1C low (black; n = 272) and high (red; n = 120) expression group (log-rank test; *p* = 0.0091; Hazard ratio (HR) = 1.64; confidence interval 1.13–2.38), based on gene chip expression data. **E** RNA-seq-based expression data of PIP5K1C in paired breast cancer (red; n = 112) and normal adjacent tissue (green; n = 112) (*p* = 0.0258; Mann–Whitney-U-Test). **F** SUM159 and MDA-MB-231 cells were transiently transfected with miR-4649-5p or mimic control and treated with 10 μM of the PIP5K1C inhibitor UNC3230 or DMSO as vehicle control. Effects on cell growth were analyzed in WST-1 assays (n = 6; mean ± SD; **p ≤ 0.01, ***p ≤ 0.005, compared by One-way ANOVA and Tukey multiple comparison test)

In order to evaluate whether targeting PIP5K1C can explain the tumor-suppressive phenotype of miR-4649-5p overexpression, we investigated the effect of siRNA-mediated PIP5K1C knockdown (Additional file 2: Fig. S4A and S4B) on TNBC cell growth. We observed reduced growth in WST-1 assays upon PIP5K1C knockdown and reduced numbers of colonies in colony formation assays (Fig. 5A, B). These results phenocopy the effects of miR-4649-5p overexpression (Fig. 2A, B) and outline a potential oncogenic role of PIP5K1C in TNBC. To further assess this possibility, we performed Kaplan Meier survival analyses. High PIP5K1C protein levels were associated with a poor prognosis in a breast cancer cohort including all subtypes (log-rank test; *p* = 0.0097; HR = 3.72; confidence interval 1.28–10.8) (Fig. 5C). While high PIP5K1C mRNA levels did not significantly correlate with overall survival of TNBC patients (log-rank test; *p* = 0.1; HR = 1.98; confidence interval 0.86–4.54), they were associated with significantly worse relapse-free survival in a large patient cohort of 392 TNBC patients (log-rank test; *p* = 0.0091; HR = 1.64; confidence interval 1.13–2.38) (Fig. 5D). Moreover, expression analysis of PIP5K1C in invasive breast carcinomas from publicly available RNA-seq data revealed that PIP5K1C exhibits significantly higher expression in cancerous tissue than in paired healthy breast tissue (n = 112 per group; *p* = 0.0258; Mann–Whitney-U-Test; Fig. 5E). Based on these findings, we evaluated a combination of the miR-4649-5p mimic and additional PIP5K1C inhibition to explore additive anti-tumor effects. For this purpose, we used a selective pharmacologic PIP5K1C inhibitor called UNC3230 [32]. We observed that the miR-4649-5p mimic, as well as UNC3230, separately had growth-reducing effects to a similar extent and that combination of both significantly enhanced this effect (Fig. 5F).

### MiR-4649-5p reduces AKT activation by targeting PIP5K1C and potentiates the growth-reducing effect of pharmacologic AKT inhibition

**Fig. 6:**
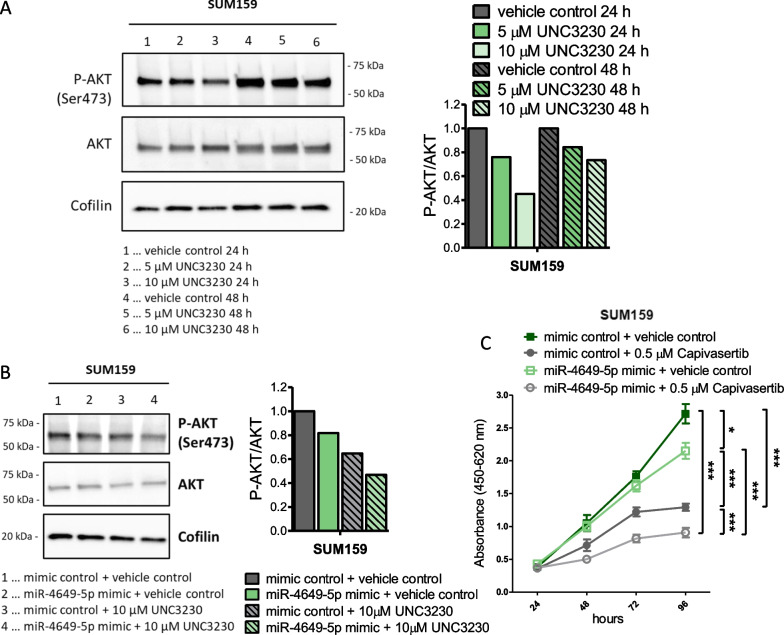
MiR-4649-5p upregulation and PIP5K1C inhibition reduce AKT phosphorylation, potentiating growth reduction by AKT inhibitor capivasertib. **A** Representative Western Blot of SUM159 cells treated with increasing concentrations of the pharmacologic PIP5K1C inhibitor UNC3230 or DMSO as vehicle control, for either 24 or 48 h (on the left), and the quantification of the detected Pospho-AKT (Ser473) normalized to the housekeeper Cofilin and total AKT (on the right). **B** Representative Western Blot of SUM159 treated with 10 nM miR-4649-5p mimic or mimic control in combination with 10 μM UNC3230 or DMSO as vehicle control for 24 h (on the left), and the quantification of the detected Pospho-AKT (Ser473) normalized to the housekeeper Cofilin and total AKT (on the right). **C** SUM159 cells were transiently transfected with miR-4649-5p or the mimic control and treated with 0.5 μM of the AKT inhibitor capivasertib or DMSO as vehicle control. Effects on cell growth were observed in a WST-1 assay (n = 6; mean ± SD; *p ≤ 0.05, ***p ≤ 0.005, compared by One-way ANOVA and Tukey multiple comparison test)

To further elucidate the mechanism behind the impact of miR-4649-5p and PIP5K1C on cell growth, we examined protein kinase B (AKT) signaling, which lies downstream of PIP5K1C and is known to have a central role in promoting cell growth and proliferation [33]. UNC3230 reduced AKT phosphorylation/activation in SUM159 cells in a dose-dependent manner (Fig. 6A). Moreover, combination of PIP5K1C inhibition and miR-4649-5p upregulation reduced AKT phosphorylation further than either alone (Fig. 6B), which may explain the additive growth reduction of UNC3230 and miR-4649-5p mimic.

As ectopic miR-4649-5p upregulation and pharmacologic PIP5K1C inhibition showed additive effects, we hypothesized that a combination of miR-4649-5p upregulation and pharmacologic AKT inhibition might result in similar potentiating effects. This is of particular interest because, while the PIP5K1C inhibitor UNC3230 is not approved for the use in breast cancer, AKT inhibitors like the pan-AKT inhibitor capivasertib have previously shown promising results in clinical trials on breast cancer patients [34–36]. We could show that the combination of capivasertib and miR-4649-5p mimic transfection increased the sensitivity of SUM159 cells to the AKT inhibitor and resulted in a stronger growth reduction than either treatment alone (Fig. 6C). In summary, these findings highlight that higher miR-4649-5p levels could make TNBC cells more vulnerable to therapeutic approaches targeting the PIP5K1C/PI3K/AKT signaling axis.

### MiR-4649-5p upregulation causes broad downregulation of pathways involved in tissue morphogenesis and biosynthesis

MiRNAs generally have broad cellular effects as they can influence a multitude of factors and whole signaling networks, both in direct and indirect ways [5]. Therefore, to gain a more comprehensive overview of the effects of miR-4649-5p, we performed a Gene Ontology (GO) enrichment analysis of the RNA-seq data which revealed that among the genes that were significantly downregulated by miR-4649-5p, there was an enrichment for genes involved in gland morphogenesis, morphogenesis of branching epithelium and branching structures, and tissue morphogenesis, as well as for genes positively associated with transcription and biosynthesis (Additional file 1: Table S4). These downregulated processes are generally considered to be oncogenic in breast cancer [37, 38], thus further corroborating the tumor-suppressiveness of miR-4649-5p.

## Discussion

Triple negative breast cancer (TNBC) is a particular burden due to its aggressive phenotype and the limitations of effective therapeutic options [2, 3]. We thus aimed to identify a novel target involved in TNBC that would allow us to better understand the disease and could serve as a rationale for the development of new therapeutic approaches. To this end, we characterized a novel microRNA (miRNA) called miR-4649-5p [13]. MiRNAs constitute pivotal cellular components as they are regulating and fine-tuning post-translational gene expression on a comprehensive and pathway-spanning scale [5, 7]. Thus, the deregulation of miRNAs can unbalance a multitude of cellular processes and contribute to disease development which is why they have received a lot of attention in cancer research [39]. Targeting miRNAs for therapeutic purposes, either upregulating or inhibiting them, has become a valid strategy for various types of cancer [20, 21].

In the present study, we discovered that miR-4649-5p exhibits a very low expression in different TNBC cell lines and that higher expression is connected to better overall survival of patients with TNBC. This led us to believe that miR-4649-5p could have a potential tumor-suppressive role in TNBC and might thereby carry potential as an RNA therapeutic. We thus examined the effect of miR-4649-5p upregulation on TNBC cell growth in vitro by using a synthetic miRNA mimic, an approach that is also applied in clinical miRNA trials [21]. We observed reduced growth, colony formation, and proliferation when cells were transfected with the miR-4649-5p mimic. Additionally, we investigated the possibility of cell death induction but did not find any indication of increased apoptosis. This suggests that the reductions in cell and colony numbers were primarily due to decreased proliferation, a therapeutic concept well-known from CDK4/6 inhibitors approved for the therapy of endocrine-dependent breast cancer [40]. Moreover, upregulation of miR-4649-5p also affected cell migration in vitro, which is interesting in regard to the fact that migratory behavior is an essential prerequisite for cancer cells to be able to initiate a metastatic cascade [41].

In order to understand the tumor-suppressive functions of miR-4649-5p that we had uncovered, we performed a whole transcriptome analysis. As miRNAs induce downregulation of their direct mRNA targets, we focused on genes that were significantly downregulated by the miR-4649-5p mimic. A Gene Ontology (GO) enrichment analysis showed that in this list of downregulated genes there was an enrichment of genes positively involved in biological processes like morphogenesis, RNA transcription, and biosynthesis. Particularly in the breast, cellular phenotypes of increased plasticity as caused by epithelial to mesenchymal transition (EMT) are not only associated with morphogenesis, for example during gland development, but are also closely connected to tumorigenesis [37]. And an overall increase in RNA transcription as well as an increased synthesis of biomolecules like nucleotides, amino acids, and lipids is associated with the demands of highly proliferative cancer cells [38, 42]. The finding that these processes are generally downregulated by miR-4649-5p substantiates the tumor-suppressive properties of miR-4649-5p. Furthermore, zooming in on the top downregulated genes, we found that 8 out of the top 10 protein-coding genes were previously reported to have oncogenic functions in different cancer types. For example, the second most downregulated candidate, *SYNPO*, coding for synaptopodin, has been discovered to be a transcriptional target of early growth response factor 4 (EGR4) and to contribute to the growth of small cell lung cancer [43]. *MTA1*, which presented the third most downregulated protein-coding gene in the RNA-seq analysis and which codes for the metastasis-associated 1 protein, is, as the name already implies, linked to epithelial-mesenchymal transition, invasion, and metastasis, amongst others in breast cancer [44, 45]. Further candidates in the top 10 were *PACS1* (phosphofurin acidic cluster sorting protein 1), which was found to exhibit increased expression in cervical cancer whereas an expression reduction led to reduced proliferation [46], and *MED16* (mediator complex subunit 16), which has been shown to promote the proliferation of ER-positive breast cancer [47].

The candidate that struck us as the most interesting and promising one was the 7th most downregulated protein-coding gene, namely *PIP5K1C*, which codes for phosphatidylinositol-4-phosphate 5-kinase type 1 gamma. PIP5K1C was of interest to us because, first of all, its 3′ UTR was predicted to contain 2 potential binding sites for miR-4649-5p and, secondly, it plays a crucial role in cells as it generates the versatile lipid signaling molecule phosphatidylinositol 4,5-bisphosphate (PI4,5P_2_ or PIP_2_) by phosphorylation of phosphatidylinositol 4-phosphate (PI4P) [30, 31]. PIP_2_ is involved in regulating cytoskeleton dynamics by activating various actin-binding proteins that drive actin polymerization [48]. In addition, it binds to focal adhesion proteins, thus linking the cytoskeleton to focal adhesion, and controls focal adhesion turnover [48, 49]. By these means, PIP_2_ promotes cell motility, migration, and cell adhesion [48, 49]. For example, Peng et al. reported that PIP5K1C is recruited to the leading edge of the plasma membrane where the subsequent increase in PIP_2_ facilitates integrin-mediated cell adhesion, which was responsible for increased migration, invasion and metastasis of hepatocellular carcinoma [50]. A study by Li and colleagues showed that a knockdown of a PIP5K1C splice variant called PIPKIγ90, an isoform that is specifically targeted to focal adhesion points via talin [51], significantly reduced the in vitro migration and invasiveness of different TNBC cell lines [52]. Another study reported similar results, namely reduced migration and also reduced growth of breast cancer cell lines upon PIP5K1C knockdown [53]. In addition, the authors discovered a correlation between high PIP5K1C expression and poorer breast cancer patient survival [53]. PIP5K1C and its product PIP_2_ have furthermore been implicated in endocytosis and protein trafficking [48, 54, 55]. For example, a splice variant of PIP5K1C that localizes to endosomes was found to promote trafficking of E-cadherin into the lysosomal degradation pathway [55]. Loss of E-cadherin is a major aspect of epithelia-to-mesenchymal transition, a central step of metastatic dissemination [56].

In summary, the above-discussed studies are in line with our findings on the oncogenic role of PIP5K1C in TNBC which help to explain the vice versa tumor-suppressive effects of miR-4649-5p that we observed. We saw that upregulation of miR-4649-5p, which entails PIP5K1C downregulation, caused decreased growth, colony formation, and migration. We could also phenocopy the effects on growth and colony formation by directly knocking down PIP5K1C. By combining miR-4649-5p upregulation with pharmacologic PIP5K1C inhibition we were able to enhance the growth-reducing effect even further. We believe that this outcome can be explained by the fact that miRNAs generally fine-tune expression, meaning miR-4649-5p downregulates PIP5K1C only to a certain extent while the remaining protein pool still fulfills its function. In combination with the PIP5K1C inhibitor though, the kinase activity of the remaining pool is inhibited, facilitating a stronger growth-reducing effect. Further highlighting the oncogenic role of PIP5K1C, we discovered PIP5K1C to show higher expression in breast cancer tissue than in healthy adjacent tissue and to be associated with worse patient survival, which was also reported by one of the discussed studies [53].

While the impact of miR-4649-5p and PIP5K1C on migration may be explained by the role of the direct PIP5K1C product PIP_2_ in actin dynamics and cell adhesion as described above, the effects on TNBC growth may be due to another downstream lipid second messenger called phosphatidylinositol 3,4,5-trisphosphate (PI3,4,5P_3_ or PIP_3_). PIP_3_ is generated from PIP_2_ by phosphoinositide 3-kinase (PI3K) and is involved in regulating cell growth, survival, and apoptosis [30, 31]. For example, PIP_3_ recruits protein kinase B (AKT) to the cell membrane, facilitating its subsequent activation by phosphorylation [57, 58]. Once activated, AKT phosphorylates and regulates downstream targets, thereby promoting cell cycle progression and survival [57, 58]. The PI3K/AKT pathway is frequently mutated and overactive in many cancer types, also in TNBC, in fact even more than in other breast cancer subtypes [59, 60]. MiR-4649-5p upregulation and PIP5K1C inhibition were able to reduce AKT phosphorylation in the SUM159 cells, confirming that the PI3K/AKT pathway lies downstream of the miR-4649-5p target PIP5K1C, which may explain the growth and proliferation-reducing effects of miR-4649-5p. In line with this, we could show that the ectopic upregulation of miR-4649-5p can make TNBC cells more sensitive to pharmacologic approaches inhibiting PIP5K1C or AKT activity. While for PIP5K1C there are no approved therapeutic interventions, AKT inhibition has previously shown promising results in various clinical trials with TNBC patients, either in combination with chemotherapy alone or in triple-combinations with chemotherapy and anti-PD-L1 [34]. For example, in the PAKT trial, the addition of the AKT inhibitor capivasertib to paclitaxel was able to significantly improve overall and progression-free survival of metastatic TNBC patients compared to paclitaxel alone [61]. It would thus be worthwhile to also explore therapeutic combinations of miR-4649-5p upregulation and AKT inhibition in in vivo settings in the future.

To conclude, we discovered that the ectopic upregulation of the otherwise very low expressed miRNA miR-4649-5p exerts tumor-suppressive effects on TNBC cells in vitro and that this may in part be ascribed to its direct target PIP5K1C, which is known to regulate migration, as well as PI3K/AKT signaling further downstream, which influences growth and proliferation. We also discovered evidence showing that increasing miR-4649-5p levels could present a therapeutic option to make TNBC cells more vulnerable to approaches inhibiting the PIP5K1C/PI3K/AKT signaling axis, thus potentiating the effect of clinical AKT inhibitors like capivasertib.

### Supplementary Information

**Additional file 1.** Supplementary Tables.

**Additional file 2.** Supplementary Figures.

**Additional file 3.** RNA-seq Data.

## Data Availability

The authors declare that all data and material used and analyzed in this study are contained within the paper and supplementary files or are otherwise available upon request.
