# Supplementary material for: MiR-4649-5p acts as a tumor-suppressive microRNA in triple negative breast cancer by direct interaction with PIP5K1C, thereby potentiating growth-inhibitory effects of the AKT inhibitor capivasertib
Source: Breast Cancer Res. 2023 Oct 6;25:119. doi: 10.1186/s13058-023-01716-2 (PMC10559525; doi:10.1186/s13058-023-01716-2)
Supplement: Supplementary file 1 — Additional file 1. Supplementary Tables. [file 13058_2023_1716_MOESM1_ESM.pdf]

**Supplementary Table S1: Primer sequences**

| Gene | Primer orientation | Sequence (5'->3') |
|---|---|---|
| **PIP5K1C** | Forward | CGAATCGGATGACACGATGGG |
| | Reverse | AAGCGCTCGGCATAGAAGC |
| **GAPDH** | Forward | AAGGTCGGAGTCAACGGATTT |
| | Reverse | ACCAGAGTTAAAAGCAGCCCTG |
| **U6** | Forward | CTCGCTTCGGCAGCACA |
| | Reverse | AACGCTTCACGAATTTGCGT |

**Supplementary Table S2: Top 20 downregulated genes in miR-4649-5p mimic transfected SUM159 cells determined by RNA-seq.** The genes were ranked according to log2 fold change.

| Ensembl Gene ID | Gene Name | Log2 fold change | Adjusted p-value |
|---|---|---|---|
| **ENSG00000130590** | ***SAMD10*** | -0,996 | 0.0002234 |
| **ENSG00000171992** | ***SYNPO*** | -0,991 | 0.0005251 |
| **ENSG00000240668** | ***KRT8P36*** | -0,956 | 0.0405872 |
| **ENSG00000182979** | ***MTA1*** | -0,946 | 3.307e-14 |
| **ENSG00000175115** | ***PACS1*** | -0,941 | 0.0003574 |
| **ENSG00000175221** | ***MED16*** | -0,920 | 4.311e-06 |
| **ENSG00000188735** | ***TMEM120B*** | -0,918 | 5.985e-14 |
| **ENSG00000186111** | ***PIP5K1C*** | -0,916 | 0.0002384 |
| **ENSG00000218175** | ***AC016739.1*** | -0,914 | 0.0171539 |
| **ENSG00000266074** | ***BAHCC1*** | -0,906 | 0.0039849 |
| **ENSG00000174996** | ***KLC2*** | -0,891 | 1.864e-10 |
| **ENSG00000179632** | ***MAF1*** | -0,832 | 3.685e-10 |
| **ENSG00000135074** | ***ADAM19*** | -0,830 | 0.0311261 |
| **ENSG00000171246** | ***NPTX1*** | -0,825 | 5.164e-22 |
| **ENSG00000182158** | ***CREB3L2*** | -0,797 | 2.129e-13 |
| **ENSG00000126003** | ***PLAGL2*** | -0,793 | 1.095e-06 |
| **ENSG00000198858** | ***R3HDM4*** | -0,792 | 2.122e-10 |
| **ENSG00000130382** | ***MLLT1*** | -0,787 | 0.0003162 |
| **ENSG00000100599** | ***RIN3*** | -0,787 | 0.0297205 |
| **ENSG00000130734** | ***ATG4D*** | -0,785 | 7.972e-07 |

**Supplementary Table S3: MiR-4649-5p binding sites in the 3' untranslated region (UTR) of phosphatidylinositol 4-phosphate 5-kinase type-1 gamma (PIP5K1C) as predicted by TargetScan** (7mer-m8…exact match of target to positions 2-8 of the mature miRNA; 8mer…exact match of target to positions 2-8 of the mature miRNA followed by an 'A').

| Site number | Target position | Predicted consequential pairing of target region (top) and miR-4649-5p (bottom) | Site type | Context++ score percentile |
|---|---|---|---|---|
| **1** | Position 586-592 of *PIP5K1C* 3' UTR | `5'    ...ACUGUGGGGACAGCCCUCGCCCU...`<br>`                             \|\|\|\|\|\|\|`<br>`3'       GAGACUCUCGGGUGGGGAGCGGGU` | 7mer-m8 | 60 |
| **2** | Position 2918-2925 of *PIP5K1C* 3' UTR | `5'  ...UUGCAUCCCAGGUUCCUCGCCCA...`<br>`              \|\|\|\|     \|\|\|\|\|\|\|`<br>`3'  GAGACUCUCGGGUGGG--GAGCGGGU` | 8mer | 92 |

**Supplementary Table S4: Gene Ontology (GO) enrichment analysis of genes that were significantly downregulated by miR-4649-5p.** The table presents significantly enriched GO biological processes, the total number of genes annotated to these processes, how many of these genes were downregulated by miR-4649-5p (enriched gene number), the number of genes that would have been expected to be deregulated at random, the fold enrichment score, whether the enrichment is positive or negative (+/-), the raw p-values and the false discovery rate (FDR)-adjusted p-values.

| GO biological process | Total gene number | Enriched gene number | expected | Fold enrichment | +/- | raw p-value | FDR-corrected p-value |
|---|---|---|---|---|---|---|---|
| growth plate cartilage chondrocyte development | 5 | 3 | 0,05 | 66,06 | + | 3.89E-05 | 3.59E-02 |
| connective tissue development | 223 | 10 | 2,03 | 4,94 | + | 5.19E-05 | 3.39E-02 |
| gland morphogenesis | 106 | 9 | 0,96 | 9,35 | + | 9.90E-07 | 1.55E-02 |
| morphogenesis of a branching epithelium | 165 | 9 | 1,50 | 6,01 | + | 2.92E-05 | 3.05E-02 |
| morphogenesis of a branching structure | 175 | 9 | 1,59 | 5,66 | + | 4.52E-05 | 3.54E-02 |
| tissue morphogenesis | 568 | 16 | 5,16 | 3,10 | + | 8.12E-05 | 4.90E-02 |
| regulation of lipid localization | 167 | 9 | 1,52 | 5,93 | + | 3.19E-05 | 3.13E-02 |
| regulation of proteolysis | 743 | 21 | 6,75 | 3,11 | + | 5.70E-06 | 2.24E-02 |
| regulation of metabolic process | 6754 | 93 | 61,34 | 1,52 | + | 2.18E-06 | 1.14E-02 |
| regulation of macromolecule metabolic process | 6214 | 86 | 56,44 | 1,52 | + | 6.37E-06 | 2.00E-02 |

| | | | | | | | |
|---|---|---|---|---|---|---|---|
| positive regulation of transcription by RNA polymerase II | 1259 | 28 | 11,43 | 2,45 | + | 1.88E-05 | 2.27E-02 |
| positive regulation of DNA-templated transcription | 1715 | 33 | 15,58 | 2,12 | + | 4.77E-05 | 3.40E-02 |
| positive regulation of nucleic acid-templated transcription | 1715 | 33 | 15,58 | 2,12 | + | 4.77E-05 | 3.56E-02 |
| positive regulation of RNA biosynthetic process | 1721 | 33 | 15,63 | 2,11 | + | 4.97E-05 | 3.39E-02 |
| positive regulation of nitrogen compound metabolic process | 3174 | 53 | 28,83 | 1,84 | + | 9.37E-06 | 2.10E-02 |
| positive regulation of metabolic process | 3858 | 61 | 35,04 | 1,74 | + | 7.66E-06 | 2.00E-02 |
| positive regulation of biological process | 6304 | 89 | 57,26 | 1,55 | + | 1.41E-06 | 1.11E-02 |
| positive regulation of macromolecule metabolic process | 3542 | 57 | 32,17 | 1,77 | + | 9.51E-06 | 1.86E-02 |
| positive regulation of cellular biosynthetic process | 2048 | 39 | 18,60 | 2,10 | + | 1.18E-05 | 1.86E-02 |
| positive regulation of biosynthetic process | 2087 | 39 | 18,96 | 2,06 | + | 1.49E-05 | 2.12E-02 |
| positive regulation of macromolecule biosynthetic process | 1943 | 36 | 17,65 | 2,04 | + | 4.35E-05 | 3.59E-02 |
| regulation of signal transduction | 2989 | 50 | 27,15 | 1,84 | + | 1.52E-05 | 1.98E-02 |
| regulation of response to stimulus | 4034 | 60 | 36,64 | 1,64 | + | 6.02E-05 | 3.78E-02 |
| regulation of cell communication | 3369 | 53 | 30,60 | 1,73 | + | 4.07E-05 | 3.55E-02 |
| regulation of signaling | 3381 | 54 | 30,71 | 1,76 | + | 2.55E-05 | 2.86E-02 |
| organonitrogen compound metabolic process | 5013 | 73 | 45,53 | 1,60 | + | 1.07E-05 | 1.86E-02 |

| | | | | | | | |
|---|---|---|---|---|---|---|---|
| nitrogen compound metabolic process | 6710 | 87 | 60,94 | 1,43 | + | 8.30E-05 | 4.82E-02 |
