# Supplementary material for: MiR-4649-5p acts as a tumor-suppressive microRNA in triple negative breast cancer by direct interaction with PIP5K1C, thereby potentiating growth-inhibitory effects of the AKT inhibitor capivasertib
Source: Breast Cancer Res. 2023 Oct 6;25:119. doi: 10.1186/s13058-023-01716-2 (PMC10559525; doi:10.1186/s13058-023-01716-2)
Supplement: Supplementary file 2 — Additional file 2. Supplementary Figures. [file 13058_2023_1716_MOESM2_ESM.pdf]

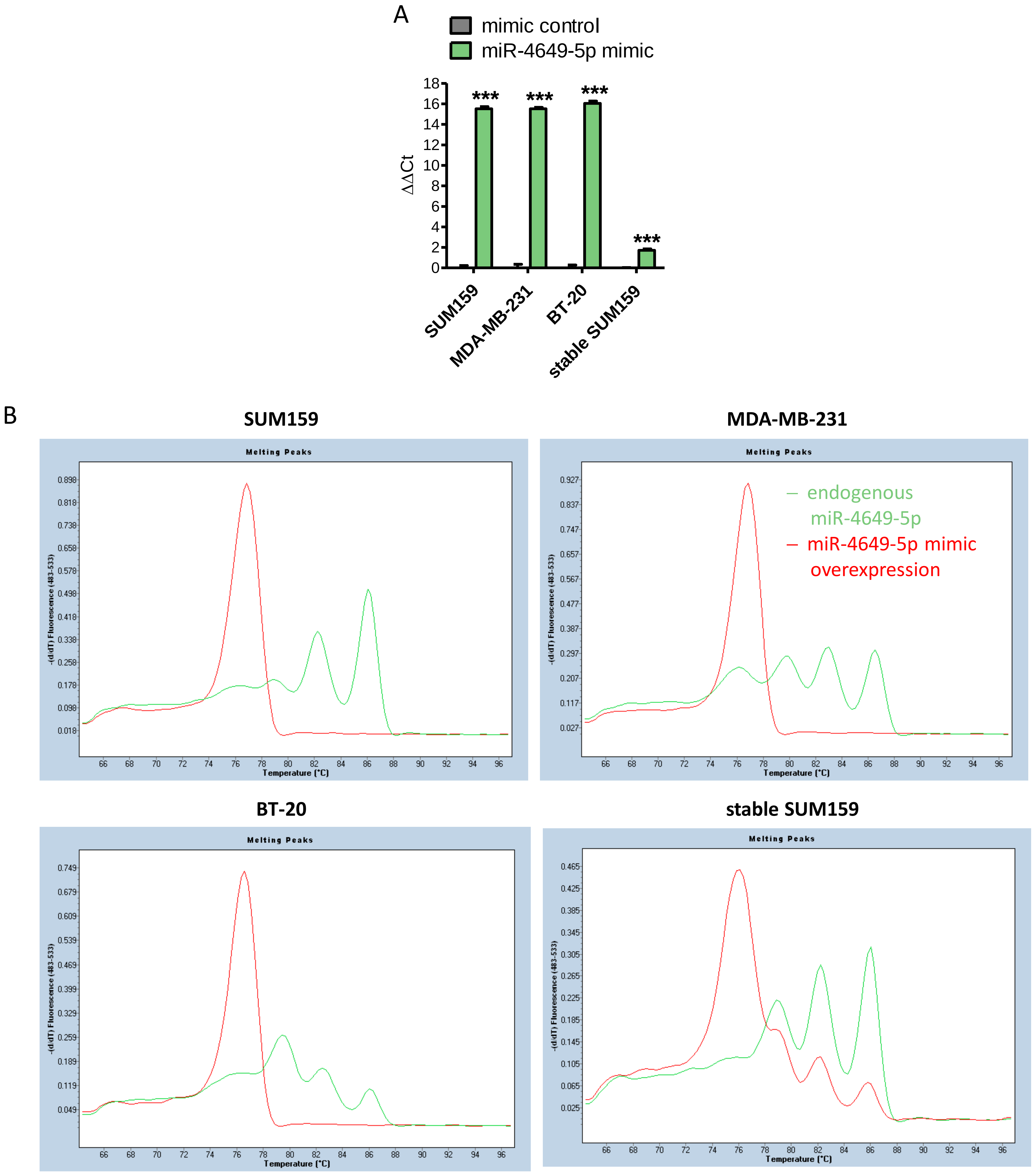

Supplementary Figure S1. Syber Green-based quantitative PCR assays detecting miR-4649-5p in three TNBC cell lines. (A) Transient overexpression of miR-4649-5p in three TNBC cell lines (48 h after transfection) and a stable overexpression quantified by qPCR (n = 3; means ± SD; ***p ≤ 0.005). **(B)** Melting curves showing the qPCR-based detection of endogenous miR-4649-5p (in green) and miR-4649-5p overexpression (in red) as positive controls. While miR-4649-5p mimic overexpression is giving a single peak of the specific miRNA product with a melting temperature of around 77 °C, the low levels of endogenous miR-4649-5p mostly result in unspecific amplifications and only very low specific amplification.

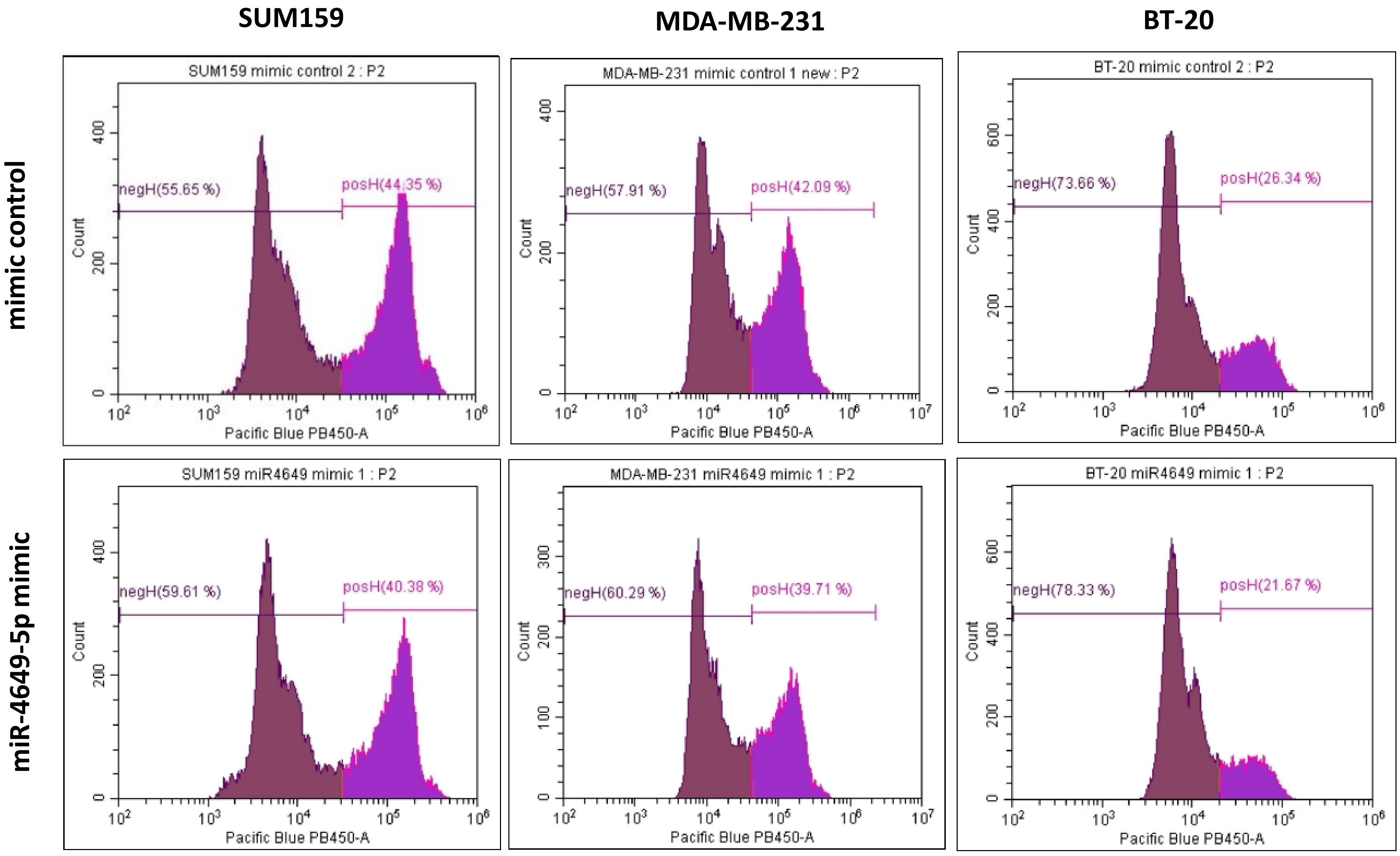

Supplementary Figure S2. Impact of miR-4649-5p on the proliferation of TNBC cell lines. Representative histograms of flow cytometric EdU proliferation assays showing the distribution and gating of EdU negative and positive cells in three TNBC cell lines transiently transfected with either mimic control or miR-4649-5p mimic for 72 h.

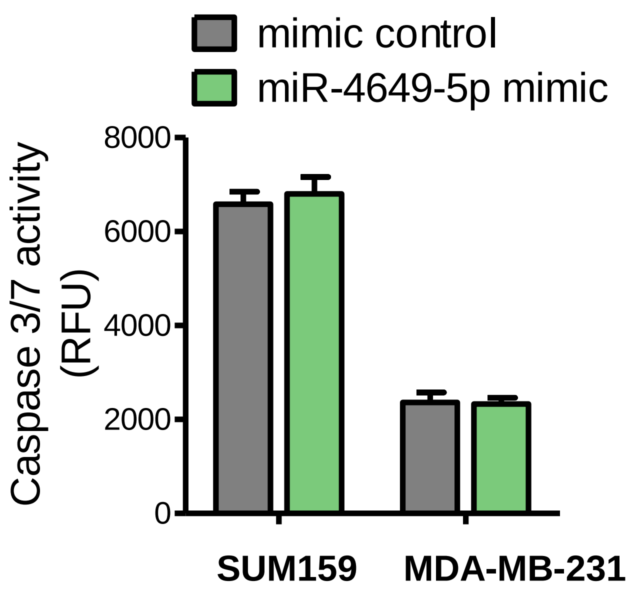

Supplementary Figure S3. MiR-4649-5p does not induce apoptosis in TNBC cell lines. Apoptosis induction was measured by a luminescent caspase 3/7 activity assay in SUM159 and MDA-MB-231 cells 48 h after miR-4649-5p mimic or control transfection (n = 4; means ± SD; RFU = Relative fluorescence units).

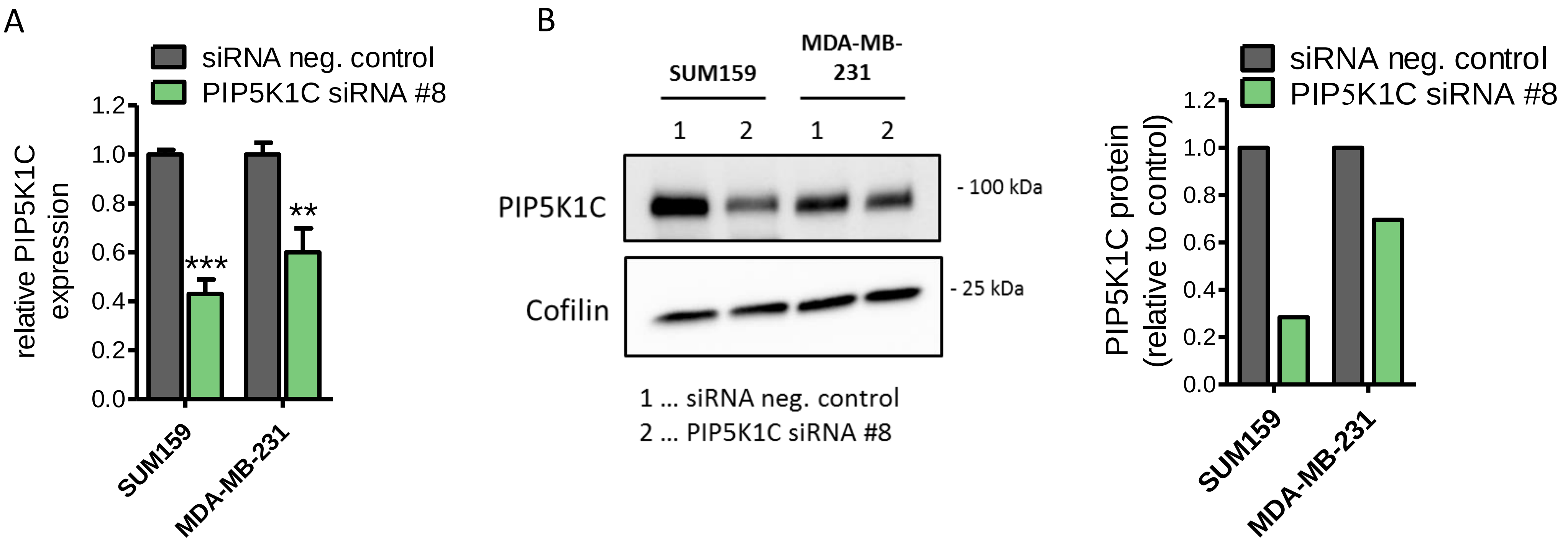

Supplementary Figure S4. Knockdown efficiency of PIP5K1C in TNBC cell lines. (A) siRNA-mediated knockdown efficiency of PIP5K1C in SUM159 and MDA-MB-231 cells as determined by RT-qPCR 48 h after transfection (n = 3; **$p \leq 0.01$, ***$p \leq 0.005$). **(B)** siRNA-mediated knockdown efficiency of PIP5K1C in SUM159 and MDA-MB-231 cells 48 h after transfection as determined by Western Blotting, showing a representative blot on the left and densitometric quantification of the blot on the right.
